# Traitement chirurgical mini-invasif d’une fracture vertébrale par compression avec atteinte bi-pédiculaire: rapport de cas

**DOI:** 10.11604/pamj.2022.42.259.36516

**Published:** 2022-08-08

**Authors:** Nicolas Pointet, Ludmilla Bazin, Benjamin Augereau

**Affiliations:** 1 Department of Orthopaedic Surgery, University Hospital, Poitiers, France

**Keywords:** Mini-invasive, ostéosynthèse, fracture vertébrale, percutanée, cas clinique, Minimally invasive, osteosynthesis, vertebral fracture, percutaneous, case report

## Abstract

Les fractures par compression vertébrale représentent une part importante de la traumatologie quotidienne en chirurgie du rachis. Leur prise en charge est codifiée grâce aux différentes classifications dont nous disposons. L´association d´une fracture tassement et d´une atteinte bi-pédiculaire de la même vertèbre conduit généralement à une prise en charge chirurgicale lourde. L´objectif principal de ce rapport de cas est de partager notre expérience d´une technique d´ostéosynthèse mini-invasive réalisée sur ce type de fracture. Le patient, âgé de 61 ans, était tombé d´un toit de 3,5 m de haut. Cliniquement, il n´avait pas de déficit sensitivo-moteur. Il présentait une douleur dorsale à 8/10 sur une échelle visuelle analogique (EVA). La tomodensitométrie a révélé une fracture par compression de la 4^e^ vertèbre lombaire (L4) de type A.3 selon la classification de l´AO. Il y avait également une atteinte bi-pédiculaire de L4. Il a bénéficié d´une prise en charge chirurgicale mini-invasive consistant en une kyphoplastie associée à une ostéosynthèse intra-pédiculaire percutanée bilatérale isolée. Nous avons observé une sédation rapide de la douleur avec une EVA de 2/10 au premier jour post-opératoire et de 0/10 à 3 mois. La consolidation osseuse était obtenue à 3 mois au scanner. A 18 mois, il n´y avait pas de signe de déplacement secondaire du matériel. L´équilibre sagittal et frontal était satisfaisant. Le patient avait retrouvé un état clinique semblable à celui d´avant l´accident. L´objectif de ce cas était de proposer une alternative chirurgicale moins invasive pour la gestion des fractures vertébrales par compression associée à une atteinte bi-pédiculaires. Dans notre cas, la combinaison d´une kyphoplastie avec une ostéosynthèse intra-pédiculaire percutanée bilatérale isolée a permis une récupération rapide après la chirurgie et un retour à l´état antérieur à l´accident.

## Abstract (en)

Vertebral compression fractures represent an important part of daily trauma in spine surgery. Their management is codified thanks to the different classifications available to us. The combination of a compression fracture and bi-pedicular involvement of the same vertebra usually leads to extensive surgical management. The main objective of this case report is to share our experience with a minimally invasive osteosynthesis technique performed on this type of fracture. The patient was 61 years old and fell from a 3.5 m high roof. Clinically, he had no sensory-motor deficit. He presented back pain at 8/10 on a visual analogue scale (VAS). The Computed tomography scan revealed a compression fracture of the 4^th^ lumbar vertebra (L4) type A.3 according to the AO classification. There was also bi-pedicular involvement of L4. He underwent minimally invasive surgical management consisting of a combination of kyphoplasty with percutaneous isolated bilateral intra-pedicular osteosynthesis. We observed a rapid sedation of the pain with a VAS of 2/10 at the first day and 0/10 at 3 months. Bone healing was achieved at 3 months on computed tomography (CT) scan. At 18 months, there was no evidence of secondary displacement of the material. Sagittal and frontal balance was satisfactory. The patient had returned to a clinical state like that prior to the accident. The aim of this case was to propose a less invasive surgical alternative for the management of bi-pedicular vertebral compression fractures. In this case, a combination of kyphoplasty with percutaneous isolated bilateral intra-pedicular osteosynthesis resulted in a rapid recovery after surgery and a return to the pre-accident condition.

## Introduction

Les fractures vertébrales par compression représentent une part importante de la traumatologie quotidienne [1,2]. Les différentes classifications établies permettent de classer ces fractures afin de standardiser leur prise en charge [3,4]. Ces classifications s´appuient, entre autre, sur la présence d´une atteinte ou non du mur postérieur [3,4], sur le calcul de la cyphose local (CL) et de la cyphose régionale (CR) ainsi que de l´angle régional traumatique (ART). Un ART > 10° est en faveur d´une prise en charge chirurgicale afin d´obtenir une meilleure réduction et une stabilisation efficace. En ce qui concerne les fractures avec un ART < 10°, il n´y a, en revanche, pas de consensus quant à la meilleure stratégie thérapeutique. Le choix d´un traitement chirurgical ou orthopédique est donc décidé en fonction des différentes caractéristiques de la fracture et du patient. Une atteinte des pédicules associée à une fracture en compression est rare mais entraine une instabilité vertébrale menant le plus souvent à une prise en charge chirurgicale extensive afin de stabiliser la colonne vertébrale [5,6]. L´objectif principal de ce rapport de cas est de partager notre expérience sur une technique d´ostéosynthèse mini-invasive réalisée sur un patient présentant une fracture vertébrale par compression associée à une atteinte bi-pédiculaire de celle-ci.

## Patient et observation

Il s´agissait d´un patient âgé de 61 ans, charpentier à la retraite ayant chuté d´un toit d´une hauteur de 3,5m.

**Résultats cliniques**: l´examen clinique ne mettait pas évidence de déficit sensitivo-moteur. Les réflexes ostéotendineux étaient présents de manière bilatérale et symétrique. Il n´existait pas de troubles vésicaux sphinctérien. Le patient présentait une douleur lombaire à 8/10 sur une échelle visuelle analogique (EVA). La douleur était majorée par la palpation et la mobilisation.

**Figure 1:**
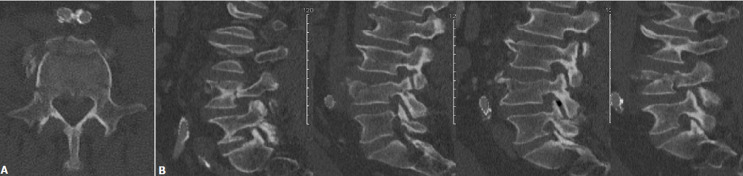
A) TDM pré-opératoire en coupe axiale, B) TDM pré-opératoire en coupe para-sagittale allant du pédicule gauche au pédicule droit

**Démarche diagnostique**: le patient a bénéficié d´une tomodensitométrie (TDM) du rachis thoraco-lombaire. La TDM retrouve une fracture par compression de la 4^e^ vertèbre lombaire (L4) de type A. 3 selon la classification de L´AO [4]. Il existait également une atteinte bi-pédiculaire de cette vertèbre (Figure 1). La CL était de 1,5°, la CR était de -20° et l´ART était de -13°.

**Intervention thérapeutique**: le patient a donc bénéficié d´une prise en charge chirurgicale sous anesthésie générale. Il était installé en décubitus ventral sur cadre. Deux amplificateurs de brillance étaient installés simultanément, un de face et un de profil. L´ensemble de la procédure a donc été réalisé sous contrôle radiologique. Deux courtes incisions de 1 cm ont été réalisées en regard des pédicules de L4. Un trocart de Jamshidi a été introduit dans chaque pédicule. La réduction fracturaire a été réalisée grâce à deux ballonnets de kyphoplastie. Des tiges guides ont ensuite été introduites dans les trocarts de Jamshidi puis nous avons retiré les trocarts. Nous avons ensuite réalisé une ostéosynthèse intra-pédiculaire grâce à la mise en place de vis pédiculaires canulées. Notre dernier temps opératoire a consisté en une injection de ciment au travers des vis canulées. Ce dernier temps a permis de stabiliser la réduction initialement obtenue par les ballonnets et d´augmenter la solidité de l´ostéosynthèse intra-pédiculaire. Cette intervention a donc permis une réduction de la fracture par kyphoplastie et une stabilisation de la fracture bi-pédiculaire par ostéosynthèse percutanée mini-invasive. Le temps de chirurgie était de 31 minutes. Le saignement était non quantifiable.

### Suivi et résultats des interventions thérapeutiques

**Figure 2:**
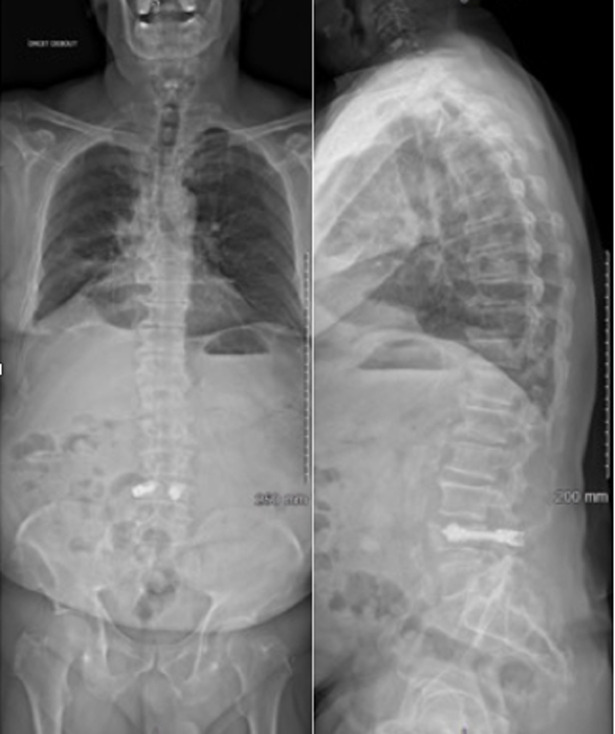
EOS post-opératoire de face et de profil

***A* *J1 post-opératoire***: l´EVA était de 2/10, la mobilisation et la mise en charge était possible. La radiographie de type EOS (Figure 2) mettait en évidence une CL de -0,5°, une CR de -25° et un ART de -8°. La Lordose lombaire L1/S1 (LL) était de 43° pour une LL théorique à 53°, l´incidence pelvienne (IP) était de 50°. La SVA était de 75mm à noter une fuite minime de ciment au niveau du plateau supérieur de L4, sans conséquence clinique. Le patient a été autorisé à sortir du service à J1. Il a bénéficié d´un arrêt de travail de 3 mois et de séances de kinésithérapie à réaliser à partir du 21^e^ jours post-opératoire.

**Figure 3:**
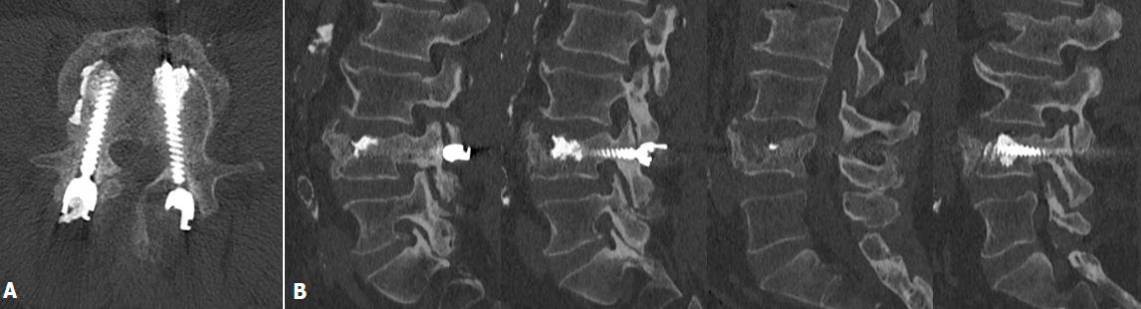
A) TDM à 3 mois post-opératoire en coupe axiale, B) TDM à 3 mois post-opératoire en coupe para-sagittale allant du pédicule gauche au pédicule droit

***A 3 mois***: L´EVA était à 0/10. Il ne présentait pas de douleur à la palpation, ni à la mobilisation. Il a arrêté les séances de kinésithérapie car il n´en ressentait plus le besoin. La TDM met en évidence une consolidation complète de la fracture du corps vertébrale ainsi que des pédicules de celle-ci, sans mobilisation secondaire de matériel (Figure 3) Il a été autorisé à reprendre toutes ses activités sans restriction.

**Figure 4:**
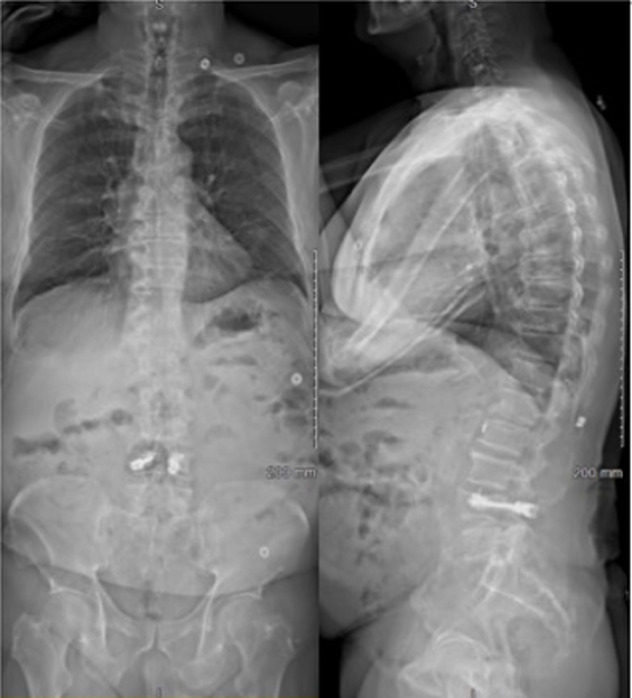
EOS à 9 mois post-opératoire de face et de profil

***A 18 mois:*** L´EVA était toujours à 0/10. Il n´existait toujours pas de douleur à la palpation, ni à la mobilisation. Il avait repris le bricolage et remontait de nouveau sur les toits. L´EOS (Figure 4) met en évidence une CL de -1°, une CR de -30°, un ART de -3°. La Lordose lombaire L1/S1 (LL) était de 56° pour une LL théorique à 53°. La SVA avait diminué à 13mm. Après 18 mois de suivi, Il existait donc une disparition complète des douleurs et un retour à l´état antérieur à l´accident. Il existait, aussi, une rééquilibration complète du rachis avec récupération d´un équilibre sagittal satisfaisant.

**Point de vue du patient**: le patient était totalement satisfait de cette intervention. Il était étonné de la rapidité de la sédation de la douleur en post-opératoire et de la simplicité des suites opératoires. Il a retrouvé une vie totalement similaire à celle qu´il avait avant l´intervention.

**Consentement éclairé**: le patient a été informé du rapport de cas, de la raison pour laquelle son cas était unique et de l´intérêt des auteurs à publier son cas.

**Consentement du patient**: le patient a donné son accord pour que ses images et autres informations cliniques soient rapportées dans le journal. Le patient comprend que son nom et ses initiales ne seront pas publiés.

## Discussion

Le but de ce rapport de cas est de présenter une approche différente sur la prise en charge des fracture bi-pédiculaire par compression. La littérature actuelle est plutôt en faveur d´une prise en charge chirurgicale invasive. La réalisation d´une ostéosynthèse s´étendant aux vertèbres sus et sous-jacent est le plus souvent recommandé [5,6]. Malheureusement, une ostéosynthèse étendue favorise l´augmentation des contraintes sur les disques intervertébraux libres aux extrémités du montage. De plus, ces chirurgies obligent la réalisation de multiple incision pour les prises en charge percutanées ou d´une incision plus extensive si elle est réalisée en ouvert. Le temps opératoire, le risque infectieux et les rsiques de complications post-opératoire sont par conséquent augmentés. Le temps d´hospitalisation et de récupération sont également plus important après ces chirurgies. Dans notre cas, la réalisation d´une chirurgie mini-invasive percutanée, avec seulement 2 incisions centimétriques, a permis de minimiser le temps opératoire. Cette prise en charge a également permis une sédation des douleurs en post-opératoire immédiat et un retour à domicile dès J1. Une rééducation a pu être réalisée de manière précoce afin d´obtenir une récupération musculaire optimal permettant ainsi de récupérer un équilibre sagittal satisfaisant. Un retour à la vie active a pu être rapidement favorisé. Il n´est toujours pas retrouvé de complication après 18 mois de suivi.

## Conclusion

Le but de ce cas était de proposer une alternative chirurgicale moins invasive pour la prise en charge des fractures vertébrales bi-pédiculaire par compression. Dans ce cas une kyphoplastie associée à une ostéosynthèse percutanée intra-pédiculaire par vissage isolé a permis une récupération rapide après chirurgie. Néanmoins, une cohorte plus importante de patient reste nécessaire pour évaluer et valider la reproductibilité de cette technique.
